# Attack Classification Schema for Smart City WSNs

**DOI:** 10.3390/s17040771

**Published:** 2017-04-05

**Authors:** Victor Garcia-Font, Carles Garrigues, Helena Rifà-Pous

**Affiliations:** Internet Interdisciplinary Institute (IN3), IT, Multimedia and Telecommunications Department, Universitat Oberta de Catalunya, Rambla del Poblenou 156, 08018 Barcelona, Spain; cgarrigueso@uoc.edu (C.G.); hrifa@uoc.edu (H.R.-P.)

**Keywords:** anomaly detection, information security, intrusion detection, security information and event management, smart cities, wireless sensor networks

## Abstract

Urban areas around the world are populating their streets with wireless sensor networks (WSNs) in order to feed incipient smart city IT systems with metropolitan data. In the future smart cities, WSN technology will have a massive presence in the streets, and the operation of municipal services will be based to a great extent on data gathered with this technology. However, from an information security point of view, WSNs can have failures and can be the target of many different types of attacks. Therefore, this raises concerns about the reliability of this technology in a smart city context. Traditionally, security measures in WSNs have been proposed to protect specific protocols in an environment with total control of a single network. This approach is not valid for smart cities, as multiple external providers deploy a plethora of WSNs with different security requirements. Hence, a new security perspective needs to be adopted to protect WSNs in smart cities. Considering security issues related to the deployment of WSNs as a main data source in smart cities, in this article, we propose an intrusion detection framework and an attack classification schema to assist smart city administrators to delimit the most plausible attacks and to point out the components and providers affected by incidents. We demonstrate the use of the classification schema providing a proof of concept based on a simulated selective forwarding attack affecting a parking and a sound WSN.

## 1. Introduction

In the coming decades, cities are facing new challenges characteristic of contemporary societies: population aging, reduction in energy consumption and carbon emissions, the struggle for greater sustainability, economic growth, etc. In addition, migratory movements are rapidly increasing the size of cities. Nowadays, 50% of the world’s population lives in cities, and it is foreseen that by 2050, this percentage will be around 70% [1].

To address these challenges, smart city initiatives have emerged proposing new ways of looking at development and city management. Generally, smart city projects have the goal of improving metropolitan infrastructure planning, automatizing urban operations, reducing costs, increasing city competitiveness, opening new business lines, creating employment and enhancing transparency and openness [2].

From a technological point of view, information systems are being deployed to transform infrastructure management towards a data-driven approach following four basic building blocks: data, analysis, feedback and adaptability [3]. In order to feed these information systems, wireless sensor technology and, more generally speaking, the Internet of Things (IoT) are used to collect data from the streets and send them to the smart city data centers. For instance, WSNs are used to manage parking lots [4] and lighting infrastructure [5].

In this context, cities are often outsourcing to external providers the installation and maintenance of their necessary WSNs. Normally, providers select technologies and implement communication protocols that are more suitable to their specific use cases, not taking into account the holistic requirements of the smart city. Thus, smart cities become highly heterogeneous and distributed environments, in which deploying standard security measures is complex, and sometimes, it is even unfeasible. Moreover, security data are hard to obtain in many cases because networks and devices are normally operated by external providers. This results in open cyber security problems affecting the smart city infrastructure [6].

Hence, in addition to the local security countermeasures that WSN providers implement in their networks, smart city administrators require systems and methodologies to discover and manage security incidents from a global perspective. Although this has been widely studied in enterprise environments, the security administration of smart cities cannot be handled in the same way, and another approach is required. At this point, smart city administrators are overwhelmed by the large amount of data, from diverse devices, multiple services, different providers, etc., and at the same time, they are not granted access to all of the necessary data and devices required to perform an adequate security analysis.

In order to improve smart city security, increase the reliability of the data obtained with WSNs and ease their subsequent analysis, this article presents two main contributions. On the one hand, we propose a framework designed to analyze smart city data combining two intrusion detection engines: a rule-based detection engine and an anomaly-based detection engine. Offering these two types of detection engines, administrators can discover known and unknown attacks. Moreover, as shown in more detail below, a security information and event management (SIEM) system is the base of the proposed framework. In this way, the framework is capable of gathering, indexing and processing the large amount of data sent by providers to the smart city central servers.

On the other hand, we introduce guidelines to manage the alarms generated by the two detection engines. These guidelines provide administrators with a procedure to identify the most likely attacks, the compromised devices and providers. Additionally, combining and correlating alarms from the two types of detection engines improves the detection results obtained only using a single engine, and it allows discovering malfunctions and attacks that have avoided the security measures installed in the WSNs by the providers.

The proposals presented in this paper make no assumption regarding specific network configurations, available countermeasures, installed IDS, etc. The framework and the guidelines presented in the paper are designed to be easily adaptable to many smart city scenarios.

The rest of this paper is structured as follows: Section 2 briefly describes the general architecture of many smart city projects designed to integrate data from several WSNs, presents the main WSN security issues and reviews the most common intrusion detection techniques. Section 3 presents an overview of the proposed framework and the alarm types that can be triggered with this framework and, in general, with similar intrusion detection platforms suitable in this context. Section 4 contains the proposed guidelines to classify alarms in order to identify the attack type, the compromised devices and providers. Section 5 shows an experimental demonstration with two types of urban WSNs as a proof of concept of the benefits of the proposed classification schema. Finally, Section 6 concludes the paper.

## 2. Background and Related Work

This section includes the background and related work. Firstly, we present the basic smart city architecture focusing on the way these systems acquire sensor data (Section 2.1). Secondly, we provide some details of WSN technology and its main security problems (Section 2.2). Finally, we give an overview of intrusion detection techniques applicable to this problem (Section 2.3).

### 2.1. Smart Cities

Cities, private companies and other institutions are already involved in smart city projects to provide solutions to the contemporary challenges that cities are facing. From a technological point of view, smart city systems are often designed as service-oriented architectures (SOA) that can be divided into three conceptual layers. The first layer includes the elements that collect information from the city (e.g., sensors, surveillance cameras, social networks, citizen complaint applications, supervisory control and data acquisition (SCADA) systems). The second layer acts as a middleware, which provides the city with an API to connect the elements of the first layer to the services offered in this layer. Among others, these services include relational and non-relational storage, geographic information systems, data analysis, cloud computing, natural language processing, business intelligence or open data. Finally, the third layer is an application layer, in which the city council and third parties implement applications based on data and services offered by the middle layer. For instance, projects that can be framed in these three conceptual layers are: the PlanIT Urban Operating System [7,8], Ubiquitous city (u-city) [9,10] and SmartSantander [11].

**Figure 1:**
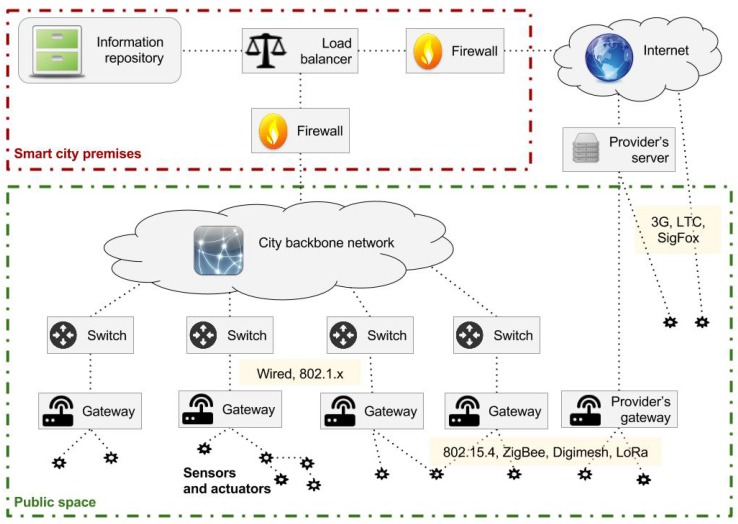
WSN data collection infrastructure.

The communication channel between street sensors and smart city central servers is represented in Figure 1. As shown in the figure, some WSNs are also equipped with actuators, which can be operated from the central servers with a downlink transmission or triggered by other first layer systems using machine-to-machine (M2M) communication. For example, vehicle detection sensors embedded in the asphalt send information to traffic controllers installed in traffic lights [12]. Nevertheless, principally, the infrastructure shown in Figure 1 is designed to collect information generated by sensors and send it to the city servers. The information flow in this schema begins in the sensors, which gather data about their environment and then send them to a gateway. Gateways finally deliver sensor data to the smart city premises. The next section will review the main characteristics of WSNs.

### 2.2. Wireless Sensor Networks

This section includes a review of WSN technology. Section 2.2.1 gives a general overview, and Section 2.2.2 introduces the security problems of this technology.

#### 2.2.1. General Overview

Wireless sensor networks are networks that communicate using wireless technology, where nodes, also known as motes, are equipped with one or several sensors to capture information about their environment.

In smart cities, it is common for motes to have autonomous cooperative communication to send values read by their sensors to a device at the edge of the WSN known as the gateway or base station. Gateways are equipped with several communication interfaces with the aim of transmitting WSN data to the smart city data centers through a conventional and reliable network (e.g., the Internet).

Wireless personal area networks (WPAN), which are the type of wireless sensor networks that are normally used to transmit sensor data, are considered especially vulnerable. Relevant technologies included in this category are ZigBee [13] and Low power Wireless Personal Area Networks (6LoWPAN) [14]. These are made of low-power devices, low-throughput and short-range communication modules, which rely in many cases on multi-hop capabilities to build an extensive network and deliver packets from the most remote nodes to the base station. Besides, motes are frequently battery powered and, therefore, are designed also with restringed processing capacity to save power. Therefore, in this paper, we focus on this type of WSN to perform attacks and intrusion analysis. However, results are generalizable to the other types of WSN.

**Figure 2:**
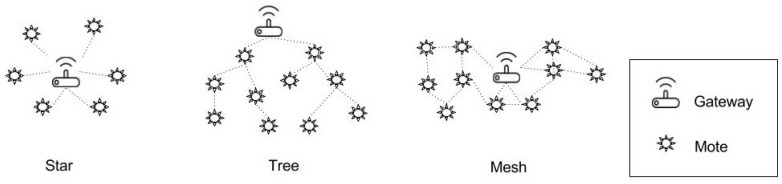
WSN communication topologies.

In WSNs, multi-hop capabilities allow three basic topologies shown in Figure 2: star, tree and mesh. In these topologies, we can see three types of nodes: gateways, motes with routing capabilities and leaf motes. Gateways and routing nodes consume large amounts of energy forwarding packets, and thereby, they are generally plugged into the electrical grid. However, leaf nodes can be battery powered, because their sole responsibility is sensing the environment and sending their own packets towards the gateway.

#### 2.2.2. WSN Security

The constrained capabilities of WSNs nodes in terms of processing power and battery are the main obstacle in deploying standard protection mechanisms and in logging security events in the nodes. Therefore, this technology is usually considered to be easy to attack. In [15,16], the authors survey the most popular attacks on WSNs, and they also describe several countermeasures to prevent them. Table 1 lists the most popular attacks and shows the basic information security principles (i.e., data confidentiality, integrity availability and non-repudiation) that can be compromised by the attacks.

Many researchers have proposed countermeasures embedded in the sensor nodes to prevent most of the attacks shown in Table 1 [15,16]. In this context, adequate embedded algorithms not only have to improve network security, but also have to consider the energetic constraints of the WSNs. Aware of this fact, researchers propose security and fault-tolerant mechanisms having energy efficiency as an additional requirement. For instance, [17] proposes a low energy consuming mechanism to select the most trustworthy cluster head for cooperative spectrum sensing environments. In [18], the authors increase the reliability in mesh networks by reconstructing a reduced amount of broken links in order to recompute faulty paths. The authors claim that the proposed algorithms use less control packets and are more energy efficient than other comparable solutions. In [19], the authors propose a secure multipath routing scheme, which is tolerant to faults and intrusions, and it also preserves the energy of the sensors.

Although these techniques are recommendable and should be implemented by the WSN providers, they cannot be considered as a security solution that smart city administrators can use to successfully manage WSN security in a holistic and generalizable manner. Indeed, the available security protocols are only applicable to certain technologies with specific requirements. For this reason, smart city administrators should approach security from a wider perspective and take into account intrusion detection techniques in order to analyze WSN data received from the providers and verify that the networks are operated correctly.

### 2.3. Intrusion Detection

Misuse detection and anomaly detection are the two basic types of techniques normally used in intrusion detection applications. On the one hand, misuse detection searches for known attack patterns in security logs. These patterns, popularly known as signatures, are defined using rules that are recorded in large databases, which, in many cases, are publicly available [20,21]. These rule-based detection systems are normally highly reliable and do not trigger a high number of false alarms. In smart cities, simple rules can monitor certain WSNs that send data in regular intervals or where sensor readings fall within clearly-defined thresholds. However, this is not the case of numerous services, and the city is a dynamic environment, which requires additional detection techniques.

On the other hand, anomaly detection identifies data that do not follow their typical behavior. Using anomaly detection, security practitioners are able to notice attacks where data are modified beyond their normal boundaries. In [22], the authors survey popular anomaly detection techniques used in a wide range of domains. Following, the most relevant techniques for the problem described in this article are highlighted:

- Tukey’s method [23] is popular for computing boundaries from a numeric univariate dataset.
- Autoregressive models [24], such as autoregressive integrated moving average (ARIMA): These models are based on the assumption that each value is somehow correlated with the previous recorded values. In this way, autoregressive models use previous values to predict future values within a confidence interval. The lower and higher boundary in the interval can be used as thresholds to point out anomalies. Autoregressive models are very common in time series analysis.
- Support vector machines (SVM) have successfully been used in [25,26] to detect intrusions. One-class SVM (OC-SVM) are a semi-supervised (i.e., labeled data are not required to train the models) version of this machine learning technique. OC-SVM are capable of training models to identify if new samples belong to the same class as the training samples.

As mentioned before in this section, in the literature, there are techniques to prevent and detect attacks in WSNs. However, as far as we know, there are no studies that analyze and propose solutions for the specific open problems related with the WSN security in a smart city, such as:

- Smart city administrators have limited access to network devices and data, which can be necessary for a complete security management. The implementation, maintenance and control of security protocols are the responsibility of external providers in many cases.
- Generic security solutions able to handle highly heterogeneous and distributed environments, with multiple systems with different technologies and security requirements do not exist.

Moreover, there are also no studies addressing the challenge of administering the plethora of warnings and alarms of different security levels that can be generated by global intrusion detection systems of the future smart city, in which there will be many more sensors, and their deployment will cover wider areas of the city. Below, in the following sections, we propose a framework and several guidelines to ease smart city administrators’ tasks related to this challenge.

## 3. Intrusion Detection Framework

In this section, we describe the basic components of the framework that we propose to gather and process smart city WSN data. Principally, the framework includes a rule-based detection engine and an anomaly-based detection engine. As we will see in Section 5, combining these two intrusion detection methods results in an improvement of the detection rate and/or the false positive rate.

**Figure 3:**
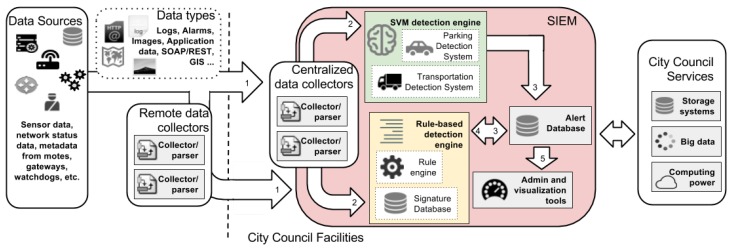
Architecture of the proposed intrusion detection framework.

Below, the steps and the components involved in the data processing through the two engines included in the proposed framework are enumerated. These are also represented in Figure 3:

1. Different sources generate a plethora of data of different types. Section 3.1 provides more details of typical fields received in the smart city data centers from WSNs. Most of the data are sent by the WSNs and collected, parsed and normalized by centralized data collectors within the smart city premises. Occasionally, remote data collectors can be required to gather the data directly from the source (e.g., to collect system logs that are not sent by the network devices).
2. At this point, two engines can access the data to detect intrusions:
  - The rule-based detection engine can correlate data from different sources. It is capable of finding patterns in the data to identify attacks that have already been reported in the literature and that are known to information security researchers.
  - The anomaly-based detection engine can warn administrators in the case of situations that do not follow the normal system behavior, even when there are no patterns matching known attacks.
  In this way, popular attacks can be easily identified and prevented, and new attacks, exploiting unknown vulnerabilities, trigger alarms that give smart city administrators the first warning signs in order to start more in-depth inquiries.
3. Alarm databases index the alarms triggered by the two detection engines.
4. The rule-based detection engine can be used again to correlate the alarms in the alarm databases and, in this way, trigger new alarms that have to be considered more reliable and having a higher priority. Section 3.2 provides more details about alarm types.
5. Administration and visualization tools provide dashboards and warning mechanisms in order to manage the system and to inform practitioners in case of an incident.

This framework is designed to be easily adaptable to different smart city architectures, and since it is deployed within the smart city central servers, it is transparent to the external providers. Thus, it can handle the high heterogeneity of the urban WSN systems. Additionally, as we will see later on, the variables used in the detection algorithms recommended in this paper are already being sent by many WSNs. In this way, our proposal not only avoids extra requirements for the providers, but also it is energy efficient, because sensor nodes do not have to send additional packets with system status variables. Preserving the energy of the nodes, which are frequently battery powered, is paramount to sensor networks in smart cities.

Moreover, as can be seen in Figure 3, the two intrusion detection engines are included in a SIEM, which have to be deployed within the city council network premises. In the enterprise context, system administrators are successfully using SIEM technology to reduce the complexity of the security administration and to automate certain tasks and security controls, such as log management, system monitoring, malware protection, vulnerability scanning, security configuration assessment and incident management [27]. Furthermore, SIEM systems offer big data collection, storage and processing services, and therefore, this guarantees the scalability of the framework’s architecture. Furthermore, data correlation and historical data management, which are generally very relevant in intrusion detection analysis, are especially efficient on this type of platform. Thus, the SIEM is a fundamental part of the proposed framework, as it becomes the centralized platform on which to process and correlate all of the information together.

The following sections study the types of data that WSN providers normally send to the smart city central servers and the types of alarms that the proposed detection engines can generate to warn smart city administrators in the case of attack.

### 3.1. WSN Data

Smart city data centers collect multiple types of data, which can be used to detect intrusions:

- Basic information about the nodes: ID, latitude, longitude, etc. The geographical position is a basic parameter to determine the area affected by the attacks.
- Basic information about the WSN: service purpose (e.g., parking, environmental monitoring), communication protocol (e.g. ZigBee, LoRa), etc. From this information, other information about the WSN can be extracted. For example, ZigBee has two possible topologies (i.e., tree and star), and its frequency bands in Europe are either 868 MHz or 2.4 GHz. This information can be used to discard possible attacks against a service and to cluster data.
- Basic information about the packets: packet number, gateway ID, timestamps, etc. Additional fields can be computed from this basic information, such as the one-way delay (OWD), which indicates the time taken by the packets from the sensor nodes to the server. This is an important field for the detection of some attacks, such as DoS, since these attacks tend to slow down packet reception.
- Basic information about system status: received signal strength indicator (RSSI), signal-to-noise ratio (SNR), etc. Some attacks have a direct impact on some of these variables. For instance, attacks generating interference impact RSSI.
- Information about the services: sensor readings, service data aggregated in time intervals, etc. These data are sent either in scheduled regular time intervals (e.g., environmental data) or when sensors have reacted to an environmental condition (e.g., parking activity). Anomalies in these data can indicate badly-calibrated sensors and data integrity attacks.
- Battery status: The objective of many WSN attacks is to exhaust sensor batteries. Therefore, this information is very useful for the detection of this type of attack.

This WSN information is accessible for the smart city administrators. Therefore, the attack models presented below are based on analyzing these types of data in order to extract the conclusions presented in this article.

### 3.2. Alarms

One of the main functions of a rule-based detection engine is to offer a way to create alarms that are triggered when a rule is fulfilled. In addition, administrators can set a severity level and an action to execute when a certain alarm is triggered (i.e., run a script). These two properties are very relevant in a system as complex as the smart city, which includes multiple subsystems and devices, and therefore, it is likely that many events end up triggering multiple alarms. Obviously, many of these alarms may not be relevant as a consequence of false alarms or ephemeral malfunctions. Hence, administrators can label the alarms with a severity code to have a first filter to distinguish the alarms that require their immediate attention and also assign an automatic action to activate a security measure.

The following subsections firstly describe the general alarm types that can be generated with the detection engines of the proposed framework. Secondly, we will focus on the alarms triggered by correlation rules, due to their important role in the identification of the attack, as we will see later on. Finally, Section 4 presents some guidelines to assist smart city administrators to interpret the different alarm types and use them to identify attacks compromising their WSNs.

#### 3.2.1. General Alarm Types

This section outlines the different types of alarms that can be triggered with the intrusion detection framework sketched in Section 3. Moreover, for each alarm type, recommendable techniques to generate the alarms are given:

- AT1, alarms triggered by simple thresholds:
  This type of alarm is triggered by simple rules that check whether a variable stays within pre-set thresholds. These thresholds can be computed manually for certain variables, for which administrators know a priori their normal boundaries; or they can be computed using methods to find outliers, such as Tukey’s method [23]. In the case of an attack that triggers an alarm of this type, administrators can have a valuable hint to find the origin of the incident, because the rule that triggers the alarm is generally associated with a single variable of a single node. However, by focusing on just one variable, several attacks may show the same effects, and therefore, other evidence needs to be gathered.
- AT2, alarms triggered by complex models:
  The alarms triggered by complex anomaly detection models allow administrators to disclose attacks that leave more subtle evidence than just single variables going over thresholds. Thus, these allow monitoring the normal state of several variables, involving different nodes and services at the same time. Compared to AT1 alarms, these alarms allow administrators to know that data are anomalous as a whole, and therefore, they have less information about the specific variables or nodes that are affected. In the article [28], we have compared several multivariate anomaly detection techniques, and we have concluded that OC-SVM is especially recommendable in this context.
- AT3, alarms triggered by time series analysis:
  Time series analysis is useful in smart cities because many services generate times series data. In these cases, new observations are closely related to previous ones. For these types of data, static thresholds are sometimes inadequate. Mathematical models, capable of predicting future values, are more suitable to point out the real values that deviate from the forecasts, which, therefore, can be considered anomalous. ARIMA models have been used in this type of analysis in various contexts, including anomaly detection in WSNs [29].
- AT4, alarms triggered by correlation:
  These types of alarms result from establishing certain relationships between an attack and its effects in an area or in some network components. These effects are in turn detected by other alarms. Therefore, this type of alarm is created to aggregate other alarms into a single one.

When system administrators create any of the alarms described above, they have to assign a severity level to the alarm. Administrators then have an easy way to filter the alarms that will actually receive their attention. Identifying the severity of the alarms is generally a specific task dependent on the specific smart city and on the security policies. For instance, administrators can flag an alarm as severe if it is implemented on an especially critical service or node; or if the alarm is implemented on a highly reliable protocol and, therefore, any sign of anomaly is a clear sign of attack. Moreover, as we will see in the next section, it is also recommended to create AT4 alarms with a high-severity level, because they are more reliable and can affect several systems.

#### 3.2.2. Alarms Triggered by Correlation Rules

A correlation rule is a type of rule that is used to group alarms that have some kind of relationship. The new alarm triggered by a correlation rule can be considered more trustworthy than the alarms that it is composed of, since it gets triggered only if several unwanted situations have already triggered some alarms. Therefore, alarms triggered by correlation rules can be considered more critical, not only because they are more reliable, but also because the alarms that it is composed of may come from several services, devices, providers, etc. Moreover, correlation rules allow administrators to reduce the number of alarms that require their attention. For instance, creating a correlation rule to group the alarms by location in a WSN reduces all of the alarms triggered by a source of interference to a single alarm, instead of receiving individual alarms from each of the sensor nodes in the area that receives the interference.

Moreover, administrators can use correlation rules to implement signatures for known attacks, for which administrators can clearly identify the attack traces. In this way, alarms triggered by these rules will not only be reliable, but they will also straightaway point to a specific attack type. The creation of correlation rules is highly dependent on the WSNs that are deployed together, their configurations and, in general, the specific scenario. Therefore, defining a methodology to create correlation rules falls out of the scope of this article.

However, general purpose correlation rules can be easily implemented in most scenarios, and as the proof of concept of Section 5 will show, this can significantly improve the detection success. As a general guideline, we recommend that smart city administrators create correlation rules to group the alarms received within a certain time interval triggered by devices sharing certain characteristics, such as a nearby location, the frequency band, the provider, the gateway, etc.

In this way, once a severe alarm built from a correlation rule is received, administrators can begin to collect more insights about the compromised components, providers, etc. In the next sections, we provide some guidelines to assist administrators to this end.

## 4. Attack Classification

In a smart city context including multiple systems deploying WSNs with hundreds of sensors, the previously described framework can trigger a plethora of alarms. Therefore, it is necessary to provide administrators with instructions on how to manage these alarms. In this section, we present a set of guidelines to classify the alarms triggered with the proposed framework into an attack model. In this way, administrators reduce the complexity at the time of analyzing the problems affecting smart city WSNs.

Firstly, Section 4.1 lists the assumptions taken regarding the smart city characteristics required to apply the proposals presented in this article. We consider that these assumptions are generic, and therefore, many current smart city models can easily adopt the guidelines presented hereafter. In Section 4.2, we propose seven different attack models based on the study of the effects that attacks have in the components of smart city WSNs in a generic way. The models include the most popular attacks against WSNs. Section 4.3 outlines a procedure with the steps required to classify the received alarms into one of the attack models. Finally, in Section 4.4, we propose a set of contingency plans to mitigate the attacks.

### 4.1. Assumptions

Below, the most relevant assumptions that we have made to create the guidelines included in this paper are listed:

- Each service uses a single WSN configuration with the same communication protocols in their nodes. If a single urban service is implemented by two providers with different networks types, in this paper, it is considered to be two different services.
- Gateways are shared by different WSNs and providers. These devices are assumed to be connected to the electricity grid, have enough computational power and a good communication network from and towards the city central servers. Therefore, gateway providers can use conventional security measures.
- The smart city is considered to be in an advanced state of development with a high density of sensors and networks. Therefore, if attack traces involve several providers, networks in several frequency bands, etc., then the analyzed scenario includes these required elements.
- Large-scale attacks are disclosed as several different attacks. For instance, a jamming attack affecting several frequency bands is considered as several jamming attacks.

### 4.2. Attack Models

**Figure 4:**
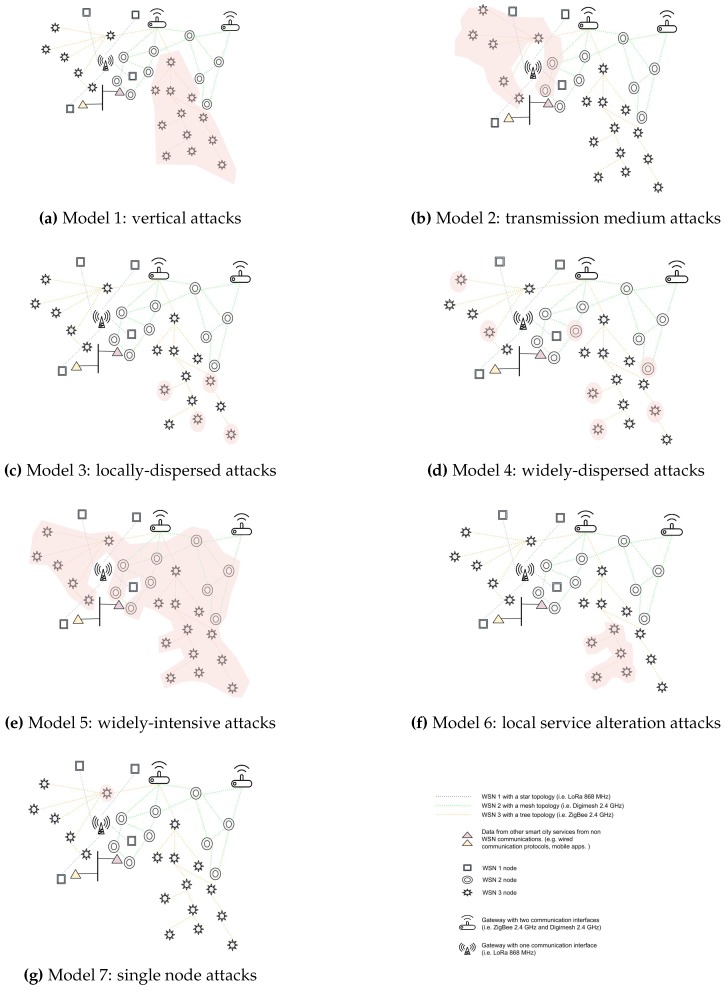
Graphical representation of the seven attack models.

Attacks in WSNs are traditionally detected analyzing particular parameters from each of the affected communication layers. Nevertheless, as previously mentioned, from the smart city administrators’ perspective, it is unrealistic to count on the availability of all of the parameters, and maintaining very specific detection systems for each WSN would be unmanageable. This section presents seven attack models, which are used later on to classify security incidents. The models in this attack schema are a general classification, based on the anomaly traces that most common attacks in WSN leave on the affected data described in Section 3.1 and on the attack’s geographical influence. For each attack model, a list of attack candidates is provided. These are the most common attacks reported in the literature (see more details in Section 2.2.2). Figure 4 shows graphical representations of the seven models. These models are described as follows:

**Model 1: Vertical attacks**
  **Description:**  Attacks that show vertical attack traces (i.e., from a group of node leaves to the base station) on a single WSN. The main aims of these attacks is to obstruct one or more paths in order to increase the arrival time of the packets from the target leaves, to crash intermediate routing nodes, to decrease node batteries or to provoke a general DoS.
  **Affected data:**  Application data, packet latency, battery level.
  **Geographical influence:**  Attack traces along large network paths starting near the leaves and ending near the base station.
  **Attack candidates:**  Path-based DoS, overwhelm, misdirection, spoof, alter or replay routing information, wormhole, Sybil to an important routing node, black hole on an important routing node, sinkhole.
**Model 2: Transmission medium attacks**
  **Description:**  Attacks that affect nearby nodes using the same frequency bands or MAC protocols. Other bands or protocols are not affected. Basically, attackers take advantage of the transmission medium in order to prevent the proper delivery or reception of packets from certain nodes. These attacks applied to routing nodes also hamper the correct communication of other nodes outside of the attacker’s direct influence area.
  **Affected data:**  Network status data (e.g., RSSI, SNR), application data, packet latency, battery level.
  **Geographical influence:**  Reduced area of nearby nodes.
  **Attack candidates:**  Unfairness, collision, jamming.
**Model 3: Locally-dispersed attacks**
  **Description:**  Attacks that affect dispersed nodes from a single WSN with the main goal of creating delays, dropping packets and depleting node batteries.
  **Affected data:**  Application data, packet latency, battery level.
  **Geographical influence:**  No geographical influence.
  **Attack candidates:**  Misdirection, spoof, alter or replay routing information, Sybil, data tampering, wormhole, selective forwarding, sinkhole.
**Model 4: Widely-dispersed attacks**
  **Description:**  Attacks that affect dispersed nodes from several WSNs. Attackers aim to reduce the proper operation of one or several WSNs. In this case, attackers do not use constant attack techniques, which would be more effective, in order to cover up their intentions and delay the moment of their discovery.
  **Affected data:**  Application data.
  **Geographical influence:**  No geographical influence.
  **Attack candidates:**  Selective forwarding at a gateway, unfairness at a gateway (not-constant), collision at a gateway (not-constant), jamming at a gateway (not-constant).
**Model 5: Widely-intensive attacks**
  **Description:**  Attacks that affect a great percentage of nodes from several WSNs using the same gateway. Attackers use these techniques to completely stop the service provided by one or more WSNs.
  **Affected data:**  Application data, battery level.
  **Geographical influence:**  Wide area of nearby nodes.
  **Attack candidates:**  Black hole at a gateway, unfairness at a gateway (constant), collision at a gateway (constant), jamming at a gateway (constant), other attacks that crash or isolate the gateway.
**Model 6: Local service alteration attacks**
  **Description:**  Attacks that affect several nearby nodes from the same WSN. The main goal is to alter application information from an area. The attackers either drop application packets or send false information.
  **Affected data:**  Application data.
  **Geographical influence:**  Reduced area of nearby nodes.
  **Attack candidates:**  Black hole, sinkhole, Sybil, data tampering.
**Model 7: Single node attacks**
  **Description:**  Attacks that aim at depleting the batteries of a single node. This becomes very critical when attackers aim at an important router node in network areas with few paths to the sink. Several of these attacks on each path divide the network.
  **Affected data:**  Battery level.
  **Geographical influence:**  No geographical influence.
  **Attack candidates:**  De-synchronization, flooding, sinkhole, collision.

### 4.3. Classification Procedure

Above, generic attack types that can compromise smart city WSNs have been shown. Below, we present a procedure to identify an attack type by analyzing the alarms generated with the detection engines explained in Section 3.

As previously seen, the proposed framework can gather a plethora of AT1, AT2 and AT3 alarms from many different nodes triggered by the same attack. As mentioned in Section 3.2.2, administrators can implement correlation rules in order to group some of these alarms and, thus, trigger a high severity AT4 alarm to warn them when the evidence of attacks is strong. Thus, the proposed classification procedure starts upon receiving an AT4 alarm, which, as previously shown, is considered reliable, and therefore, these type of alarms require administrators’ attention.

**Figure 5:**
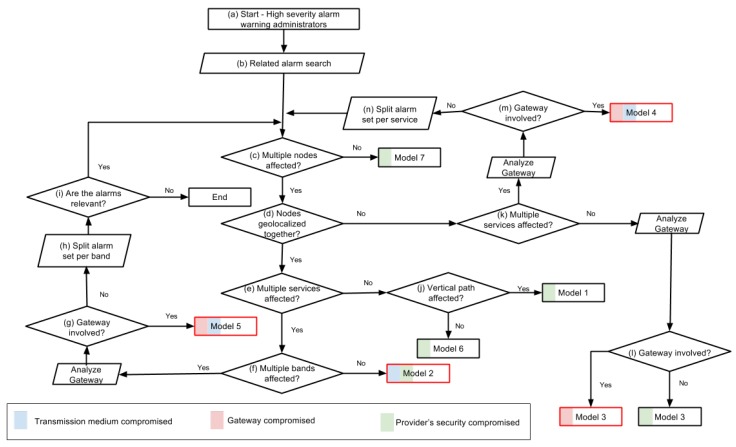
Procedure to classify attacks into seven attack models according to the evidence in the alarms triggered by the detection engines.

Figure 5 presents the workflow with the classification schema. As the figure shows, the process begins when administrators receive a high severity AT4 alarm (Step (a)). Then, administrators have to decide which other alarms registered in the system could be related to the incident and, therefore, have to be collected for analysis (Step (b)). In general, these related alarms are the ones triggered by nearby nodes during a short time interval before or after the incident, or by other components of the same network, or the same provider, etc. As a result of Step (b), administrators identify a set of alarms that is relevant for the classification of the incident. In the subsequent steps, this set of alarms is analyzed to answer the following questions:

- Are multiple nodes affected? (Step (c)).
- Are the affected nodes geolocalized together? (Step (d)).
- Are multiple services affected? (Steps (e) and (k)).
- Are multiple frequency bands affected? (Step (f)).
- Is the gateway involved in the attack? (Steps (g), (l) and (m)).
- Are the alarms in the set of alarms relevant? (Step (i)).
- Do vertical paths show signs of being affected? (Step (j)).

It is worth noting that verifying whether a gateway is involved in the attack is usually the responsibility of the gateway provider, who has full access to the gateway. Moreover, as mentioned in Section 4.1, we consider that gateways are not constrained in terms of processing power, are connected to the electricity grid and have reliable telecommunication connections. Therefore, providers can perform complete analysis on the gateways with conventional security tools (e.g., anti-virus, IDS) to test if they have been compromised.

In addition to the analysis through the aforementioned questions, the workflow eventually requires splitting the alarm set into several subsets according to a certain criterion (e.g., by frequency band) (Steps (h) and (n)). Splitting the alarm set is important to differentiate between several alarms due to different incidents occurring in the same devices around the same time. When there is no model clearly identifiable from the alarm set, as Loops (c)–(i) and (c)–(n) show, we propose an iterative process that divides the alarm set into several partitions, and each of them is used to start the classification procedure again. Thus, alarms that are not related are divided into different partitions and, therefore, analyzed separately. These unrelated alarms from the same area at the same moment in time can be due to concurrent attacks, false alarms, etc.

Lastly, the final states of the procedure in Figure 5 show the attack model that fits the evidence given by an alarm set. The states where third party services or important communication nodes are affected are indicated by a red box.

Below, in order to illustrate the classification process, an example incident is resolved:

1. A high severity alarm (AT4) calls the smart city administrators’ attention (Step (a)) to a parking WSN controlled by Provider A. This AT4 alarm is defined with a correlation rule that groups three or more AT1 alarms (triggered if the received RSSI is above a threshold) in a two-hour window. Administrators look in the system for other alarms in the same time window sharing the same area or equipment (Step (b)). They find other AT1 alarms from an environmental WSN, which is controlled by Provider B. In this case, the AT1 alarms were programmed with a simple rule that would check that the data from the sensors were received in the scheduled intervals. All of these alarms make up the alarm set used by the administrators to classify the incident.
2. At this point, administrators start analyzing the alarm set by answering the questions proposed in the schema. Firstly, they run a simple query to determine if multiple nodes are affected (Step (c)). The result of this query is positive.
3. Administrators visually inspect the nodes to figure out if the affected nodes are geolocalized together (Step (d)). The result of this query is positive.
4. Administrators verify if multiple services are affected in all the alarms (Step (e)). The result of this query is positive (because the parking service and the environmental monitoring service are affected).
5. Administrators make another simple query to check if multiple bands are affected (Step (f)). The result of this query is negative (because the WSNs from the two services are configured in the same frequency band).
6. Administrators resolve that the analyzed WSNs are under an attack from Model 2. Moreover, the affected data that triggered the alarms support this conclusion, i.e., RSSI over the limit in the parking WSN and packet latency increased in the environmental WSN. Thus, the proposed classification procedure allows administrators to conclude that this is a transmission medium attack, where the source of the attack affects nearby nodes using the same frequency bands and/or MAC protocols. In this type of attack, the affected nodes can be from different WSNs and different providers. Likely attacks are: unfairness, collision and jamming.

With this procedure, we point out the most likely attack model and the possible attacks for each model, we limit the affected nodes, the area and the providers. Additionally, we also indicate if the attack is compromising the transmission medium (i.e., blue mark), a gateway (i.e., red mark) or the provider’s infrastructure (i.e., green mark). The next section recommends contingency plans to mitigate the short- and medium-term consequences of the attacks.

### 4.4. Contingency Plans

At the end of the procedure in the previous section, the administrators have a sharper picture of the cause of the alarms, and therefore, they can contact the service providers to look for a solution to the problem. In general, solving this type of attack can be a long process, since it involves coordinating several parties and analyzing many devices, which can be difficult to access. Hence, at this moment, besides looking for a solution to patch the possible vulnerabilities, it is also paramount to mark the data from the compromised services and to avoid new data becoming compromised.

Below, basic recommendable strategies to mitigate the possible negative consequences of the attacks are listed. These contingency plans are divided into short- and medium-term actions depending on whether other services are affected.

**Short-term actions:**
  **Transmission medium compromised**
    - Data from the compromised service in quarantine
    - Data from nearby services in quarantine
    - Exclusion area required
  **Gateway compromised**
    - Data from the compromised service in quarantine
    - Data from other services in the compromised gateway in quarantine
    - If other gateways available, then compromised gateway excluded
  **Provider’s security compromised**
    - Data from all the services of the provider in quarantine
**Medium-term actions:**
  **Transmission medium compromised**
    - Data from the compromised service and the nearby services excluded from learning, statistical and analysis processes
  **Gateway compromised**
    - Data from the compromised service and other services in the compromised gateway excluded from learning, statistical and analysis processes
  **Provider’s security compromised**
    - Data from the services of the compromised provider excluded from learning, statistical and analysis processes

Putting the measures listed above in place avoids the situations where other services compromise new data and where compromised data are used in an urban operation. For instance, in the short term, we propose establishing exclusion areas when attacks compromise the transmission medium. In this way, mobile devices using WSN technology avoid entering areas where their transmissions can be in danger. In the medium term, we propose that data from compromised devices should be excluded from business analytical tasks to avoid drawing the wrong conclusions. For example, the municipality could decide to expand a parking facility, basing the analysis on compromised WSN data from a parking lot, indicating overuse.

Depending on the critical nature of the affected services, it is possible that other contingency strategies are required. Hence, administrators have to analyze the details of each use case to figure out the necessary additional countermeasures.

## 5. Proof of Concept

This section includes a proof of concept to demonstrate the use of the proposed classification schema to detect and locate a data availability attack. This type of attack is difficult to detect, can involve several services from different WSNs from different providers and requires the analysis of multiple types of data. The implemented attack in this proof of concept simulates a 20% selective forwarding attack that affects a wide area with dispersed nodes from several WSNs from different providers. Selective forwarding attacks become more obvious as the dropped packet rate and the number of affected nodes increase and when it affects a reduced and non-dispersed area. Therefore, we analyze the benefits of using the defined schema in a highly complex detection scenario.

### 5.1. Scenario Description

**Figure 6:**
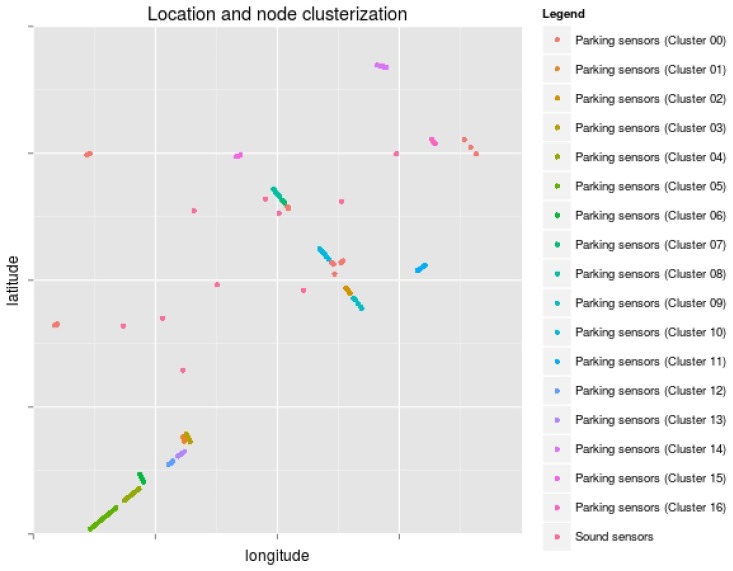
Sensor positions and division of the parking sensor nodes in clusters.

In this section, we build a scenario for the demonstration, and we analyze the data using the core packages included in R [30] and the packages e1071 [31] and caret [32] for the OC-SVM classification and FPC [33] for the clustering algorithms. The original data came from two Barcelona service providers from July–November 2015. The two services collect data from street parking and sound meters. The data from the parking service include the gateway identifier used by each packet to send the data from the parking sensors to the central servers. The sound data do not include this information. For this proof of concept, it is necessary to extend the real data to have a dataset with the minimum amount of information to be able to simulate attacks and, subsequently, perform anomaly analysis. Therefore, in order to have a proper dataset for this demonstration, the following actions are performed:

- The sound meters are placed in the area of the parking network respecting the layout of both networks. Figure 6 shows the node position in both networks.
- A gateway identifier is assigned to each packet received from the sound network. The assignment of a gateway to each sound packet is based on the gateway computational load. Thus, gateways processing many parking network packets receive fewer sound packets. As we will see later on, this gateway identifier is only used to simulate the attack. When we perform the analysis later, the gateway identifier is considered unknown in the sound packets.
- The data types described in Section 3.1 are received at the data center from diverse services and are generally considered available to any service. However, in this scenario, the sound service does not provide the sequence number of the application packets, which can be used to easily calculate the application packet loss. Therefore, the packet loss rate is added to the sound meter data.
- A normal packet loss rate (i.e., not due to attacks) is also considered. There is a wide variability on ordinary packet loss rates in WSNs, depending on several network characteristics, such as the communication protocols or the node density. In [34], the authors performed measurements using the collection tree protocol (CTP), which is one of the most popular routing protocols in WSNs, and they found end-to-end packet delivery rates from 90.5%–99.9%. Thus, in this simulation, the sound monitoring service has a conservative 90% packet delivery rate (i.e., 10% packet loss rate without attack).

In this scenario, we simulate a selective forwarding attack in one of the gateways, where 20% of the received packets are randomly dropped. This causes data loss from sensors belonging to the two services spread throughout the neighborhood. This scenario, with only two services, is a basic configuration for a smart city and demonstrates the value of the proposed classification schema. Scenarios including more services and providers would increase the complexity of the analysis; but, at the same time, more alarms from other WSNs would be triggered, and then, there would be more evidence of the attack, which would enhance the value and the results of the classification procedure.

### 5.2. Analysis

This section presents the basic procedures to detect anomalies and trigger alarms (Section 5.2.1). Thus, this proof of concept briefly shows how to apply the techniques reviewed in the previous sections. Moreover, it shows the importance of implementing correlation rules, and it demonstrates how to use the classification schema proposed in this paper (Section 5.2.2).

#### 5.2.1. Basic Detection Analysis

In this section, a basic analysis using three different intrusion detection techniques is performed. Firstly, rule-based detection, which generally triggers a low rate of false positives, is used defining a threshold (the AT1 type in Section 3.2.1) to unveil attacks affecting the sound service. Secondly, anomaly-based detection (the AT2 type in Section 3.2.1) is used to complement the previous rule-based detection, thus adding the possibility of detecting unknown attacks. Due to the excellent performance of OC-SVM in the smart city context shown in the comparative study in [28], in this proof of concept, OC-SVM is used to detect attacks on aggregated parking data. Finally, the third technique correlates the alarms triggered by the previous two detection techniques (the AT4 type in Section 3.2.1). In this way, this demonstration shows that different techniques are required to detect attacks in this type of scenario. Additionally, it shows how smart city administrators (who receive data from all of the different subsystems) are capable of improving the attack detection results in the WSNs of an area in the city, especially when they take into account that a single attack can affect at the same time different networks, communication protocols, providers, etc.

In order to perform the detection analysis, we first divide the data from this simulation into three sets. Fifty percent of the data are used for training purposes; 25% is used as a validation data to tune the parameters of the algorithms; and the last 25% is used as a test dataset. The selective forwarding attack is only applied to the validation and the test data.

**Figure 7:**
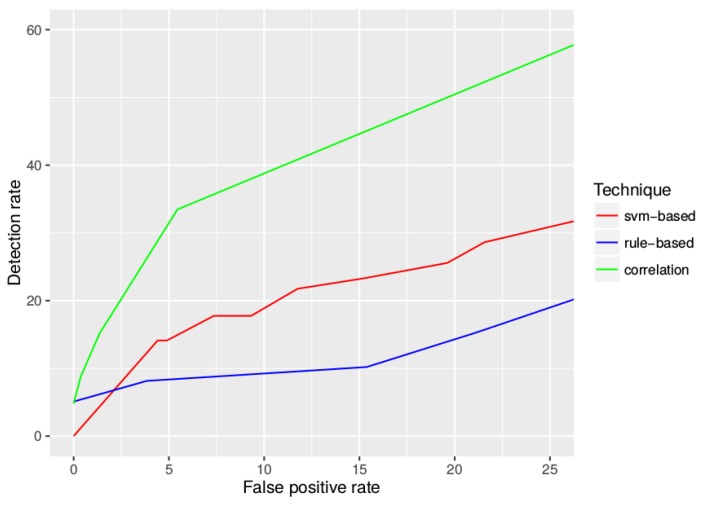
Evolution of the detection rate and the false positive rate of the three techniques. In the rule-based detection, increasing the packet loss threshold decreases the detection rate and the false positive rate (e.g. defining threshold=9 results in a 10.20% detection rate and a 15.38% false positive rate; defining threshold=10 results in a 8.16% detection rate and a 3.85% false positive rate). In the one-class (OC)-SVM detection, a higher value of the hyperparameter ν increases the detection rate and the false positive rate (e.g., defining ν=0.01 results in a 14.12% detection rate and a 4.41% false positive rate; defining ν=0.02 results in a 17.75% detection rate and a 9.31% false positive rate). In the detection by correlation, including more alarms in the correlation rule decreases the detection rate and the false positive rate (e.g., aggregating two alarms from any of the two systems results in a 33.47% detection rate and a 5.45% false positive rate; aggregating three alarms results in a 15.14% detection rate and a 1.36% false positive rate).

The three types of alarms are implemented as follows:

- We use rule-based detection on the sound data. From this service, we aggregate the number of lost application packets in one-hour windows, and we define a threshold to determine the maximum number of lost application packets that is considered normal (i.e., not due to an attack). Setting a high threshold implies decreasing the detection rate (i.e., the number of detected attacks divided by the number of total attacks) and the false positive rate (i.e., instances that are incorrectly classified as attacks divided by the number of total instances that are not attacks).
- We use OC-SVM on the parking data. In this case, the procedure to detect attacks has the following steps:
  1. We group the sensor nodes into clusters by location using DBSCAN [35]. Figure 6 shows the division by clusters.
  2. We aggregate the number of changes in the parking spots per cluster into one-hour windows.
  3. We train an OC-SVM model with the aggregated data. This model is used later to test whether new aggregated parking data have been gathered in a period when the network was working properly or when the network was under attack. In order to evaluate the detection rate considering various false positive rates, we trained various OC-SVM models selecting different values for their hyperparameters. For example, increasing the value of the hyperparameter ν, which defines the maximum fraction of outliers present in the training data, also increases the detection rate and the false positive rate.
- We create a simple correlation rule (the AT4 type in Section 3.2.1) that is triggered when various alarms from any of the previous two detection techniques have been triggered in one hour. In order to build this correlation rule, we compare the detection rate and the false positive rate of varying amounts of alarms triggered by any of the two detection engines. As shown below, by decreasing the number of required alarms in the correlation rule, we can gradually increase both the detection rate and the false positive rate. The results are shown in Figure 7.

Generally, alarms based on thresholds are more reliable than alarms triggered by machine learning techniques. If an attacker performing a selective forwarding in the gateway drops a large amount of packets, then the AT1 alarm implemented with a threshold for the number of lost application packets from the sound service would be enough in order to discover the attack. However, the most challenging situations arise when the attacks affect few sound packets and many parking packets, which is the scenario that is implemented in this proof of concept. Thus, we demonstrate the advantages of using correlation rules and the classification schema proposed in this paper.

**Figure 8:**
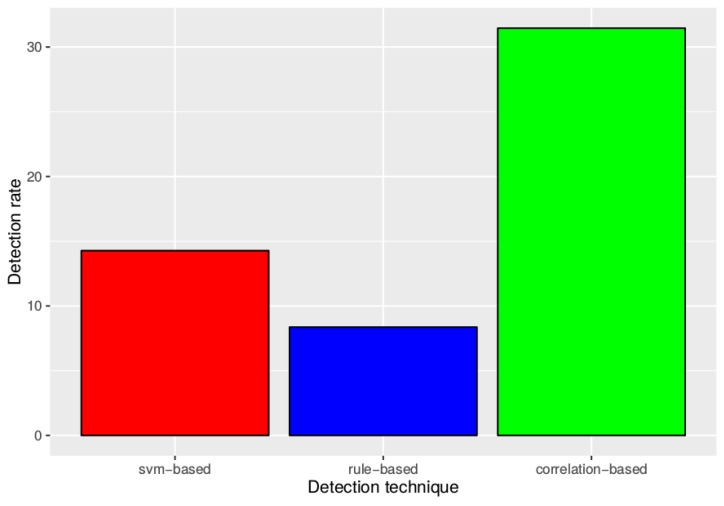
Detection rate comparison between the three techniques at a 5% false positive rate.

Figure 7 and Figure 8 show the performance of the detection techniques in this scenario. Figure 7 shows that, in general, for a false positive rate lower than 25%, the combination of alarms from the first two techniques in a correlation rule outperforms the other techniques operating separately. In the smart city context, the false positive rate must be kept low to avoid overwhelming system administrators. As seen in Figure 8, setting a maximum 5% false positive rate implies that the detection rate using correlation detection is more than 2.2-times higher than using only OC-SVM and more than 3.7-times higher than using only rule-based detection. Therefore, this outcome shows that administrators can significantly improve detection results by implementing simple correlation rules as shown in this example.

These results are particularly interesting when taking into account that the detection scenario is highly complex due to a 20% selective forwarding dropping rate (only 10% more than the normal loss rate for the sound network) and the difficulty of detecting anomalies in nodes spread in a wide area. There is also a need to detect the attacks within a one-hour window, which allows time to apply short-term contingency actions. Therefore, the performance of the proposed detection process would increase in scenarios with a higher dropping rate, where the attack is focused on a narrow area or without short-term contingency requirements.

This simulated detection process also shows that alarms triggered by the correlation rule must be considered as highly severe. The other alarms are also important to trace incidents in the smart city, but they are less reliable and provide less information about the incidents than correlation rules. In the next section, starting from the reception of a highly severe alarm, we used the proposed schema to classify the incident into an attack model.

#### 5.2.2. Enhanced Analysis with Attack Classification

**Figure 9:**
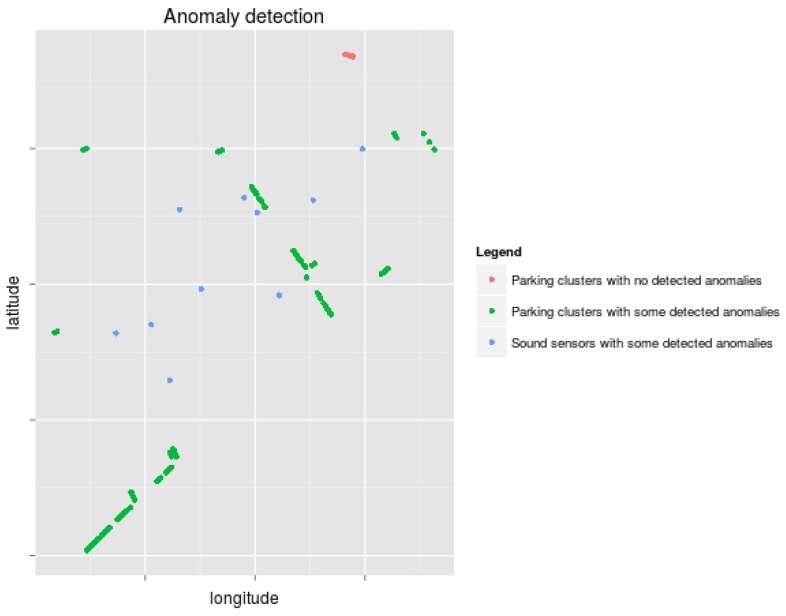
Sound sensors and parking sensor clusters where the attack has been detected.

In this proof of concept, AT1 and AT2 alarms can be constantly triggered and, therefore, do not deserve administrators’ attention until an AT4 alarm is received. Upon receiving an AT4 alarm (Step (a) in the schema shown in Figure 5), administrators can proceed with the other steps in the classification procedure:

1. Administrators must retrieve other alarms from the same service, from the same area or from some network that shares important components with the network that triggered the alarm (Step (b)). Thus, in this scenario, alarms from the parking and sound service have to be retrieved and make up the base alarm set for this analysis.
2. The answer to the question, “Multiple nodes affected?”, is “Yes” (Step (c)).
3. The answer to the question, “Nodes geolocalized together?” is “No” (Step (d)), since Figure 9 shows that the anomalies detected in the sound sensors and parking clusters are sparsely distributed.
4. The answer to the question, “Multiple services affected?” is “Yes” (Step (k)).
5. At this point, administrators could have already taken preventive countermeasures involving the transmission medium and the gateway. They should request the gateway provider to undertake a comprehensive analysis of the gateway (Step (m)) to confirm that the networks are under the attack Model 4. As can be seen in Section 4.2, this corresponds to a widely-dispersed attack, so administrators can conclude that the most likely attack candidates aim at the gateway and match the traces of a selective forwarding, a non-constant unfairness, a non-constant collision or a non-constant jamming. In this situation, following the proposed recommended contingency strategies, they should mark an exclusion area near the gateway, redirect WSN traffic to other gateways (if possible) and quarantine the data from the compromised gateway, the nearby services and other services that were using this gateway.

As this example has shown, using the classification schema and taking into account the alarms from the smart city WSNs as a whole, smart city administrators not only detect anomalies in a more reliable way, but are also provided with a clearer picture of the attacks causing the incidents.

## 6. Conclusions

Smart cities are generally managed by city councils that outsource urban data collection to specialized external providers. In many cases, considering the point of view of smart city administrators, this results in a loss of view and control on the incidents in the data collection process. The goal of this article is to ease smart city administrators’ tasks in the case of attack in the urban WSNs. Thus, in this paper, we have proposed a framework designed from the point of view of the smart city administrators to detect intrusions in the WSNs. The framework includes two types of detection engines. On the one hand, it includes a rule-based detection engine in order to detect known attacks identifiable with an attack signature. On the other hand, it includes an engine based on searching for anomalies to be also capable of detecting unknown attacks for which there are yet no implemented signatures or for which it is not feasible to implement them. Taking into account the most popular intrusion detection techniques and the way WSNs in the context of smart cities operate, we have analyzed the alarm types that can typically be triggered with these two detection engines, and we have collected the most suitable techniques to trigger each alarm type.

Furthermore, in an environment such as the smart city, system administrators have a large amount of data from diverse devices, and the intrusion detection systems usually trigger a plethora of alarms, which, in addition, are not always important and are difficult to interpret. Therefore, intrusion detection in smart city WSNs is not a straightforward task and cannot be fully automatized and treated like a black-box in which a set of anomaly detection algorithms can be used to detect any attack in any specific scenario. Administrators have to select the variables to monitor, draw conclusions in the case of alarms, create correlation rules to trigger meaningful alarms that deserve their attention, etc. Nowadays, specific protocols guiding smart city administrators on how to resolve these types of situations do not exist. Thus, in this article, as a first contribution in this research field, we have proposed a schema to classify the evidence left by attacks against smart city WSNs into seven different attack models. With this schema, we provide smart city administrators with guidelines to identify the attacks and the compromised network components. These models have been devised by taking into account the effects that the attacks have on the components of smart city WSNs in a generic way. For each attack model, we have provided a list of attack types to narrow down the most likely cause of attack, and we have provided a set of contingency plans to mitigate short- and medium-term consequences of the attacks.

This article also shows that the combined use of rule-based detection and OC-SVM by means of simple correlation rules can significantly improve detection results. Thus, the combination of the two detection engines’ output in a correlation rule can outperform the other techniques operating separately, and this has been shown in a complex detection scenario with a 20% selective forwarding dropping rate (only 10% more than the normal loss rate for the sound network). To the best of our knowledge, this is the first approach towards studying the effects of correlating WSN security analysis of different services in the smart city. The proof of concept has also exemplified the procedure to follow to figure out the most likely attacks and components compromising two WSNs.

The proposed schema does not claim to be comprehensive, and therefore, it does not include all of the possible attacks for all possible smart city configurations. This schema has been designed to be adaptable to many smart cities, and it should be treated as a guideline to develop a security and incident response system for smart city WSNs.

Therefore, as future work, it would be of great value to provide the best ways to detect attacks taking into account the specificities of the typical services included in the smart cities. Although we have already suggested certain algorithms to set thresholds up, data integrity attacks have different effects for application data in the different services, and therefore, in order to have more effective detection, it is necessary to study how attacks affect the most important variables on each service or in the network and then find the best algorithm to detect them. Moreover, the proposed guidelines to narrow down the list of candidate attacks compromising a system have to be enhanced with a methodology to build correlation rules integrating alarms from the two detection engines. Furthermore, in a real smart city, it is necessary to have an automatic system including a comprehensive list of attacks and other possible causes of network malfunctioning. The rule-based detection engine is theoretically capable of identifying any type of known attack if it leaves traces on the data. However, as far as we know, there are currently no public signature databases to identify attacks in this context.

## Figures and Tables

**Table 1:** Information security principles compromised by WSN attacks.

**Physical Layer**	
**Attack**	**Compromised Principles**
Data tampering	Integrity
Node tampering	Confidentiality, integrity, availability
Node replication	Integrity
Jamming	Availability
**Data Link Layer**	
**Attack**	**Compromised Principles**
Sybil	Integrity, availability
Interrogation	Availability
Exhaustion	Availability
Collision	Availability
Unfairness	Availability
**Network Layer**	
**Attack**	**Compromised Principles**
Sleep deprivation	Availability
Internet smurf	Integrity, availability
Misdirection	Availability
Acknowledgment spoofing	Integrity, availability
Spoofed, altered, or replayed routing information	Integrity, availability
Wormhole	Availability
Sybil	Integrity, availability
Selective forwarding and black hole	Availability
Hello flood	Availability
Sinkhole	Availability
Homing	Confidentiality
**Transport Layer**	
**Attack**	**Compromised Principles**
De-synchronization	Integrity, availability
Flooding	Availability
**Application Layer**	
**Attack**	**Compromised Principles**
Deluge	Integrity
Path-based DoS	Availability
Overwhelm	Availability
Eavesdropping	Confidentiality
Re-play	Non-repudiation

## References

[1] Naphade M., Banavar G., Harrison C., Paraszczak J., Morris R. (2011). Smarter cities and their innovation challenges. Computer.

[2] Gurdgiev C., Dirks S., Keeling M. (2010). Smarter Cities for Smarter Growth.

[3] The Royal Academy of Engineering (2012). Smart Infrastructure: The Future.

[4] Zheng Y., Cao J., Tang V.W.S. An intelligent car park management system based on wireless sensor networks. Proceedings of the IEEE 2006 First International Symposium on Pervasive Computing and Applications.

[5] Merlino G., Bruneo D., Distefano S., Longo F., Puliafito A., Al-Anbuky A. (2015). A smart city lighting case study on an openstack-powered infrastructure. Sensors.

[6] Cerrudo C. (2015). Keeping Smart Cities Smart: Preempting Emerging Cyber Attacks in U.S. Cities.

[7] Living PlanIT PlanIT Urban Operating System. http://living-planit.com.

[8] Gil-Castineira F., Costa-Montenegro E., Gonzalez-Castano F.J., López-Bravo C., Ojala T., Bose R. (2011). Experiences inside the ubiquitous oulu smart city. Computer.

[9] Lee Y.W., Rho S. U-city portal for smart ubiquitous middleware. Proceedings of the IEEE International Conference on Advanced Communication Technology (ICACT).

[10] Chen M. (2013). Towards smart city: M2M communications with software agent intelligence. Multimed. Tools Appl..

[11] Smart Santander. http://www.smartsantander.eu/.

[12] Sensys Networks, Inc. (2014). Sensys Networks VDS240 Wireless Vehicle Detection System. Design Guidelines for Intersection Applications.

[13] ZigBee Standards Organization (2012). Zigbee Specification.

[14] Montenegro G., Kushalnagar N., Hui J., Culler D. (2007). Transmission of IPv6 Packets over IEEE 802.15. 4 Networks.

[15] Kavitha T., Sridharan D. (2010). Security vulnerabilities in wireless sensor networks: A survey. J. Inf. Assur. Secur..

[16] Modares H., Salleh R., Moravejosharieh A. Overview of security issues in wireless sensor networks. Proceedings of the IEEE International Conference on Computational Intelligence, Modelling and Simulation.

[17] Chkirbene Z., Hasna M.O., Hamila R., Hamdi N. Energy-efficient based on cluster selection and trust management in cooperative spectrum sensing. Proceedings of the IEEE Wireless Communications and Networking Conference Workshops (WCNCW).

[18] Yang T., Sun Y., Taheri J., Zomaya A.Y. (2013). DLS: A dynamic local stitching mechanism to rectify transmitting path fragments in wireless sensor networks. J. Netw. Comput. Appl..

[19] Challal Y., Ouadjaout A., Lasla N., Bagaa M., Hadjidj A. (2011). Secure and efficient disjoint multipath construction for fault tolerant routing in wireless sensor networks. J. Netw. Comput. Appl..

[20] Symantec Symantec Attack Signatures. https://www.symantec.com/security_response/attacksignatures/.

[21] Bond D. Quickdraw SCADA IDS. http://www.digitalbond.com/tools/quickdraw/.

[22] Chandola V., Banerjee A., Kumar V. (2009). Anomaly Detection: A Survey. ACM Comput. Surv..

[23] Tukey J.W. (1977). Exploratory Data Analysis.

[24] Montgomery D.C., Jennings C.L., Kulahci M. (2015). Introduction to Time Series Analysis and Forecasting.

[25] Este A., Gringoli F., Salgarelli L. (2009). Support vector machines for TCP traffic classification. Comput. Netw..

[26] Kaplantzis S., Shilton A., Mani N., Sekercioglu Y.A. Detecting selective forwarding attacks in wireless sensor networks using support vector machines. Proceedings of the IEEE International Conference on Intelligent Sensors, Sensor Networks and Information.

[27] Montesino R., Fenz S., Baluja W. (2012). SIEM-based framework for security controls automation. Inf. Manag. Comput. Secur..

[28] Garcia-Font V., Garrigues C., Rifà-Pous H. (2016). A Comparative Study of Anomaly Detection Techniques for Smart City Wireless Sensor Networks. Sensors.

[29] Yu Q., Jibin L., Jiang L. (2016). An Improved ARIMA-Based Traffic Anomaly Detection Algorithm for Wireless Sensor Networks. Int. J. Distrib. Sens. Netw..

[30] R Core Team (2015). R: A Language and Environment for Statistical Computing.

[31] Meyer D., Dimitriadou E., Hornik K., Weingessel A., Leisch F. (2015). e1071: Misc Functions of the Department of Statistics, Probability Theory Group (Formerly: E1071), TU Wien.

[32] Kuhn M., Wing J., Weston S., Williams A., Keefer C., Engelhardt A., Cooper T., Mayer Z., Kenkel B., the R Core Team caret: Classification and Regression Training.

[33] Hennig C. fpc: Flexible Procedures for Clustering.

[34] Gnawali O., Fonseca R., Jamieson K., Moss D., Levis P. Collection tree protocol. Proceedings of the 7th ACM Conference on Embedded Networked Sensor Systems.

[35] Ester M., Kriegel H.P., Sander J., Xu X. (1996). A density-based algorithm for discovering clusters in large spatial databases with noise. Kdd.

